# Development, Cytotoxicity and Eye Irritation Profile of a New Sunscreen Formulation Based on Benzophenone-3-poly(ε-caprolactone) Nanocapsules

**DOI:** 10.3390/toxics7040051

**Published:** 2019-09-22

**Authors:** Thallysson Carvalho Barbosa, Lívia Éven Dias Nascimento, Cristiane Bani, Taline Almeida, Marcelo Nery, Rafael Silva Santos, Luana Renyelle de Oliveira Menezes, Aleksandra Zielińska, Ana Rita Fernandes, Juliana Cordeiro Cardoso, Alessandro Jäger, Eliezer Jäger, Elena Sanchez-Lopez, Luciana Nalone, Eliana Barbosa Souto, Patrícia Severino

**Affiliations:** 1 University of Tiradentes (Unit), Biotechnological Postgraduate Program. Av. MuriloDantas, 300, Aracaju 49010-390, Brazil; thallyscb@hotmail.com (T.C.B.); liviajere2@hotmail.com (L.É.D.N.); talinealmeida2009@hotmail.com (T.A.); marcelonery.pg@gmail.com (M.N.); rafa.au@icloud.com (R.S.S.); luanamenezespsa@gmail.com (L.R.d.O.M.); juliana_cardoso@unit.br (J.C.C.); luciana.nalone@hotmail.com (L.N.); 2 Institute of Technology and Research (ITP), Nanomedicine and Nanotechnology Laboratory (LNMed), Av. Murilo Dantas, 300, Aracaju 49010-390, Brazil; 3 Department of Morphology, Federal University of Sergipe (UFS), Avenida Marechal Rondon, São Cristovão 49100-000, Brazil; crisbani@gmail.com; 4 Department of Pharmaceutical Technology, Faculty of Pharmacy, University of Coimbra (FFUC), Pólo das Ciências da Saúde, Azinhaga de Santa Comba, 3000-548 Coimbra, Portugal; zielinska-aleksandra@wp.pl (A.Z.); anaritavfernandes@gmail.com (A.R.F.); esanchezlopez@ub.edu (E.S.-L.); 5 Institute of Macromolecular Chemistry, Academy of Sciences of the Czech Republic, v.v.i., Heyrovsky Sq. 2, 162 06 Prague, Czech Republic; alejager@gmail.com (A.J.); jagereliezer@gmail.com (E.J.); 6 Department of Pharmacy, Pharmaceutical Technology and Physical Chemistry, and Institute of Nanoscience and Nanotechnology (IN2UB), Faculty of Pharmacy, University of Barcelona, 08028 Barcelona, Spain; 7 CEB—Centre of Biological Engineering, University of Minho, Campus de Gualtar, 4710-057 Braga, Portugal; 8 Tiradentes Institute, 150 Mt Vernon St, Dorchester, MA 02125, USA

**Keywords:** polymeric nanocapsules, poly(ε-caprolactone), benzophenone-3, sunscreen, cytotoxicity, ocular tolerance

## Abstract

The objective of this work was to characterize the toxicological profile of a newly developed sunscreen formulation based on polymeric nanocapsules (NCs) loading benzophenone-3 (BZP3). NCs composed of poly(ε-caprolactone) carrot oil and Pluronic^®^ F68 were produced by emulsification-diffusion method. Their mean particle size (Z-Ave) ranged from 280 to 420 nm, polydispersity index (PDI) was below 0.37, while zeta potential (ZP) reached about |+11 mV|. No cytotoxic effects were observed in L929 fibroblast cell line for the blank (i.e., non-loaded) NCs and BZP3-loaded NCs (BZP3-NCs). The semi-solid sunscreen formulation was stable over time (centrifugation testing) and exhibited non-Newtonian pseudoplastic behavior, which is typical of products for topical application onto the skin. The sun protection factor (SPF) value reached 8.84, when incorporating BZP3-NCs (SPF of 8.64) into the semi-solid formulation. A synergistic effect was also observed when combining the formulation ingredients of nanocapsules, i.e., SPF of carrot oil was 6.82, blank NCs was 6.84, and BZP3-loaded NCs was 8.64. From the hen’s egg-chorioallantoic membrane test (HET-CAM) test, the non-irritation profile of the developed formulations could also be confirmed. The obtained results show a promising use of poly(ε-caprolactone) nanocapsules to be loaded with lipophilic sunscreens as benzophenone-3.

## 1. Introduction

Sunscreens are commonly applied onto large skin areas to prevent erythema, premature aging, and skin cancer. A suitable sunscreen formulation should exhibit bioadhesiveness when applied onto the skin without any toxicological reaction, while creating a protective film on the stratum corneum for efficacy and durability [1]. Sunscreens usually contain both chemical (e.g., organic) and physical (e.g., TiO_2_ and ZnO) filters [2]. Chemical organic filters are classified as UVA (benzophenones, anthranilates, dibenzoylmethanes, avobenzones, and ecamsule) and UVB (aminobenzoates, such as para-aminobenzoic acid (PABA) derivatives; salicylates; cinnamates; camphor derivatives; octocrylene; and ensulizole) blockers [2,3]. To avoid white marks upon application onto the skin surface, chemical UV filters are preferentially used, also because these are easier to formulate in sunscreens [4].

Among the UV filters, benzophenone-3 (BZP3) is a promising UV-blocking agent, because it can reflect and absorb a wide spectrum of UV light (200–400 nm) with a maximum absorbance of 288 and 325 nm. BZP3 has been approved by the U.S. Food and Drug Administration (FDA) and is being usually used in combination with other UV filters [5]. 

Since UV filters must be kept onto the uppermost skin regions, innovative and more effective formulations are still needed in order to meet these demands [5,6,7,8]. No systemic absorption must be ensured to limit the risk of toxicity and/or adverse side reactions. UV filters and sunscreens must be explicitly authorized by the European Parliament and the Council for cosmetic products. As BZP3 was shown to be easily penetrated into the skin causing side effects, such as photoallergies or skin irritation [9], its use has been questioned by the Scientific Committee on Consumer Safety (‘SCCS’) since 16 December, 2008 [10]. By virtue of Commission Regulation (EU) 2017/238, it has been approved up to the maximum admissible concentration of 6% (wt/wt) as a UV-filter in cosmetic sunscreen products. Benzophenone-3 (BZP3) can also be applied up to 0.5% (wt/wt) in all types of cosmetic products for UV protection, not posing risk to human health (unless allergic and photoallergic reactions are reported).

Conventional oil-in-water emulsions are commonly used in skin formulations for the delivery of anti-aging actives, as well as in sunscreen formulations [11]. However, due to the risk of penetration of actives through the skin, dermatitis and other allergic reactions may occur. Furthermore, the degradation of UV filter and cellular damage have also been reported [4]. With this in mind, nanotechnology is being singled out as the latest development in technological innovations to achieve this goal. The results released in the scientific literature demonstrate that nanoformulations can strongly impact on the action of sunscreens by providing a higher concentration of the UV filter on the stratum corneum and overcoming the disadvantages of conventional emulsions.

Polymeric nanocapsules (NCs) have been recognized to avoid the risk of photodegradation, as they reduce the risk of skin absorption of the active ingredient or may even increase the sun protection due to the occlusive barrier forming on the skin surface [12]. Due to these synergistic effects, higher effectiveness and higher safety may be achieved with a lower amount of UV filter loading the NCs. Polymeric NCs have high encapsulation efficiency for lipophilic active ingredients, which, together with a longer contact time of the formulation with the skin, an enhanced protection effect may be achieved. The use of NCs in sunscreens was shown to exhibit a lower frequency of allergic reactions and toxic effects [13,14,15,16]. 

The aim of this study was to develop a sunscreen formulation composed of polymeric NCs loading benzophenone-3 as a model active ingredient of lipophilic character. The particle size of polymeric NCs was determined by photon correlation spectroscopy (PCS) and transmission electron microscopy (TEM) and their cytotoxicity profile was checked against fibroblasts. The polymeric NCs were then formulated as a semi-solid sunscreen, for which the sun protection factor (SPF), stability, viscosity, and compatibility by hen’s egg-chorioallantoic membrane test (HET-CAM) were determined [17].

## 2. Materials and Methods

### 2.1. Materials

Tween^®^ 80 and Crodamol^®^ GTCC were provided by Croda (Brazil) and Miglyol^®^ (812 e 840) was bought from Sasol (Germany). Pluronic^®^ F68 was purchased from Sigma-Aldrich (Sintra, Portugal). Poly(ε-caprolactone) was a gift from Purac (Gorinchem, The Netherlands). The oils of natural origin were purchased from Henrifarma (São Paulo, Brazil). L929 fibroblast cell line was obtained from a cell bank in Rio de Janeiro (BCRJ) (Rio de Janeiro, RJ, Brazil). Consumables for cell culture assays were purchased from Gibco (Alfagene, Invitrogene, Portugal). Dimethyl sulfoxide, ethanol, glycerine, triethanolamine, and methyl-thiazolyl diphenyl-tetrazolium bromide (MTT) were bought from Sigma-Aldrich (Sintra, Portugal). Ammonium acryloyldimethyltaurate/vinylpyrrolidone (VP) copolymer (Aristoflex® AVC) was obtained from UPI Chem (Somerset NJ, USA). Crodamol SG^®^ was obtained from Croda (East Yorkshire, UK). Ultra-purified water was obtained from MiliQ® Plus system (Millipore, Germany). 

### 2.2. Production and Characterization of Polymeric Nanocapsules

#### 2.2.1. Production of Polymeric Nanocapsules

NCs were produced by emulsification-diffusion method [14] and selected for their high reproducibility for the synthesis of polymeric nanoparticles with limited use of toxic organic solvent [18]. In this case, the organic phase was composed of poly(ε-caprolactone), oil, and acetone. The water phase consisted of an aqueous solution of Pluronic® F68. Both the phases were separately heated until 40 °C was reached, under magnetic stirring (KASVI-K40-1820H, Brazil). Then, the organic phase was slowly added to the water phase using a syringe to produce an emulsion which was kept under stirring for 24 h. For the loading of the NCs with the active ingredient, BZP3 was added to the organic phase prior to emulsification. The composition of polymeric NCs is shown in Table 1.

#### 2.2.2. Photon Correlation Spectroscopy

The obtained NCs were characterized by photon correlation spectroscopy (Malvern Zetasizer, Nano ZS; Malvern Instruments, Worcestershire, UK) in order to assess mean particle size (Z-Ave), polydispersity index (PDI), and zeta potential (ZP). NCs were dispersed with ultra-purified water prior to the analysis of Z-Ave and PI at a fixed angle of 173° and a temperature of 25 °C. The zeta potential (ZP) was measured by laser Doppler anemometry at 25 °C in purified water adjusting conductivity (50 µS/cm) with a solution of 0.9% (m/V) NaCl. Each recorded value is the average of three measurements (mean ± SD).

#### 2.2.3. Cytotoxicity

Cell viability was tested against a L929 fibroblast cell line using the colorimetric methyl-thiazolyl-tetrazolium (MTT) assay for the assessment of cell metabolic activity. Cells were cultured in RPMI containing 10% fetal bovine serum with 50 IU/mL penicillin and 50 μg/mL streptomycin in a 5% CO_2_ atmosphere (37 °C). NCs in the concentration of 30 μg/mL were seeded in 96-well culture plates (2 x 10⁴ cells/well). A solution of MTT was placed in contact with the cells, which were incubated at 37 °C for 3 h. After removal of the MTT, dimethyl sulfoxide was placed for solubilization of the tetrazole salt crystals and then optical density reading was performed by the automated plate reader at the wavelength of 570 nm. The tests were conducted in quadruplicate and then normalized. Statistical data analyses were performed recording the mean ± standard error of the mean (SEM) and using one-way analysis of variance (ANOVA) followed by Tukey post-test. Graph Pad Prism software (version 5.0) was used. P values <0.05 were considered statistically significant.

#### 2.2.4. Transmission Electron Microscopy

Morphology of the obtained NCs was observed in a transmission electron microscope (TEM-MSC JEOL 210, USA), operating at 200 kV acceleration. In this case, samples were directly dripped onto the carbon-covered copper grid.

### 2.3. Production and Characterization of the Semi-Solid Sunscreen

#### 2.3.1. Production of the Semi-Solid Sunscreen

The semi-solid sunscreen formulation was produced by adding 1.0% (wt/v) of Aristoflex^®^ AVC in the aqueous dispersion of NCs keeping it at rest at 24 h. After that, 2.5% (wt/v) of Crodamol SG^®^, 5% (wt/v) of glycerine, 5% (wt/v) of Tween^®^ 80, and 10% (v/v) of ethanol were sequentially added under mechanical stirring (Fisatom 713D, Brazil) until reaching the desired consistency. Finally, the pH was corrected to 7.4 with triethanolamine. 

#### 2.3.2. Viscosity Analysis

The viscosity was determined by using Viscometer Fungilab (Visco Start-R Brazil). The spindle R4 was put into the semi-solid sunscreen in order to avoid the formation of air bubbles in contact with the surface of the sample. The assay was performed at shear rates ranging from 1 to 100 rpm at room temperature. The viscosity analysis for semi-solid formulations were performed in triplicate.

#### 2.3.3. Stability Tests

Macroscopic analysis was performed to visually inspect the semi-solid sunscreen for its color, homogeneity, and consistency for a period of 2 months. Then, 2 g of the semi-solid formulation was added in a tube to perform the centrifugation test. The semi-solid sunscreen was submitted to a cycle of 3000 rpm for 30 min at room temperature using a centrifugal Daiki (DTC-16000-BI, Brazil). The semi-solid sunscreen was submitted to freeze and thaw cycles. The stability test including 2 cycles was performed for a period of 7 days. In each cycle, the sample remained at a particular temperature for a period of 24 h at different temperature conditions, namely, −5 ± 2 °C, 40 ± 2 °C and −55 ± 2 °C. Finally, pH of the formulations was evaluated 7 days after production.

#### 2.3.4. Sun Protection Factor (SPF)

The SPF was determined according to the method described by Santos et al., [19]. This method is simple, fast, and easy. The absorbance of the samples was measured in the UV-B wavelength range (290–320 nm), with increments of 5 nm and three determinations were made at each point. The SPF was calculated applying the Mansur equation:

SPF “spectrophotommetric” = CF × ΣEE (λ) × I (λ) × Abs (λ)(1)

where EE(l)—erythemal effect spectrum, I(l)—solar intensity spectrum, Abs(l)—absorbance of sunscreen product, and CF—correction factor (= 10). The values of EE x I are constants.

#### 2.3.5. In Vitro Hen’s Egg Test on the Chorioallantoic Membrane (HET-CAM) 

The methodology was done as previously reported [20,21,22]. The eggs were incubated for 10 days 38 ± 0.5 °C. The membrane was then carefully removed using fine-tipped forceps and the CAM was exposed. The formulations were added on embryonated hen´s egg membrane, and their effects were studied for 300 s. As a positive control (for vascular hemorrhage and lysis), 300 μL of sodium hydroxide solution (0.1 M) was applied, while 300 μL of NaCl solution (0.9 wt%) was applied as a negative control. Then, a volume of 300 μL of the semi-solid formulations diluted in distilled water was applied to the CAM of the eggs. The assay was performed in triplicate. It was monitored for any event (hemorrhage, lysis, and coagulation) for 300 s and the time of onset of these three events was recorded. The standard irritation scores were calculated as follows:

300 [IS] = 5 [301 − H] + 7 [301 − L] + 9 [301 − C](2)

where IS—irritation score, H—start time in seconds for the onset of bleeding, L—start time in seconds to start lysis, and C—start time in seconds for coagulation to start. The tested materials were classified according to the irritability score: 0 to 0.9 = non-irritant, 1 to 4.9 = slightly irritant, 5 to 8.9 = moderately irritant, and 9 to 21 = strongly irritating.

#### 2.3.6. Morphological Analysis

The images of the semi-solid sunscreen were captured from the blades using an Olympus C-7070 video camera and an Olympus CX31 microscope [5].

## 3. Results and Discussion

The mean particle size of the developed non-loaded and BZP3-loaded poly(ε-caprolactone) NCs was recorded within the nanometer range (Table 2), with a slightly higher increase in size and PDI when loading the active ingredient (287.43 ± 13.60 nm versus 418.60 ± 56.28 nm). Monomodal size distribution reflects a homogeneous population of particles and higher long-term stability. For both the formulations, the PDI was recorded below 0.4, and the particles also showed no tendency for aggregation over time. In a sunscreen formulation, it is desirable that particles cover the stratum corneum providing occlusive effect onto the skin, without penetration into deeper layers. It is also known that the physicochemical properties of NCs, in particular their size, may influence skin penetration [23]; the higher the size, the lower the capacity for skin penetration. Smaller sized particles may enter the stratum corneum via hair follicles. De Brum et al. have shown that nanoparticles around 200 nm could reach the dermis [23]. Our results do not compromise the feasibility of aqueous dispersions for topical application as a sunscreen formulation. 

The zeta potential (ZP) is an important parameter to estimate long-term stability and it helps to predict the risk of aggregation of nanoparticles. The recorded values of ZP were +11.30 ± 0.79 mV and +6.11 ± 0.87 mV for non-loaded and BZP3-loaded NCs, respectively. The positive values are attributed to the positively-charged poly(ε-caprolactone). Additionally, Pluronic^®^ F68 has been used as a non-ionic surfactant to improve steric stabilization, thereby avoiding agglomeration. The non-ionic block co-polymer contributed for the decrease of zeta potential. In the next step, the nanocapsule was incorporated in a hydrogel also favoring stability.

For the assessment of the safety profile of sunscreen formulations, and to limit the need for animal experiments, cytotoxicity testing in fibroblast cell lines is a viable approach [24]. Utsunomiya et al. have recently reported that benzophenone-3 exhibits toxic effects in systemic use [25], which can be attributed to oxidative stress with an increase in intracellular Zn^2+^ levels. However, BZP3 is approved by FDA and Agência Nacional de Vigilância Sanitária (ANVISA) and there are commercially available formulations for its use as sunscreen. 

**Figure 1:**
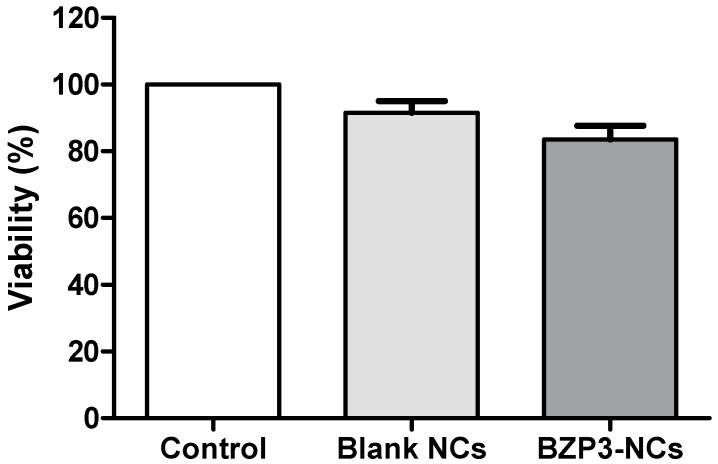
Cell viability of L929 fibroblast cell line treated with blank NCs and BZP3-NCs. Cytotoxicity at concentrations ranging from 10–150 μg/mL. Results are expressed as the mean ± standard deviation (*n* = 4).

Using the MTT assay, our non-loaded (blank) and BZP3-loaded NCs did not exhibit metabolic changes in the cell culture, nor cell death (Figure 1). Cell viability above 70 wt% were recorded (91.12 wt% for non-loaded and 89.45 wt% for BZP3-loaded NCs), which demonstrated the NCs to be non-cytotoxic [26,27,28]. No statistical difference was observed using the ANOVA test. The MTT reduction assay is widely used and was the first developed cell viability assay. Viable cells with active metabolism convert MTT reagent into a purple-stained product, while dead cells are not capable of such reaction. The mechanism behind it involves reactions with reducing molecules such as NADH that transfer electrons to MTT. The released purple-colored compound is an insoluble intracellular precipitate that accumulates near the cell wall. In this work, we did not observe any difference among samples; however, the sample may accumulate in the wall or the cell with the MTT. No cytotoxicity was observed in this assay, but more details may be obtained with complementary in vitro cell techniques.

**Figure 2:**
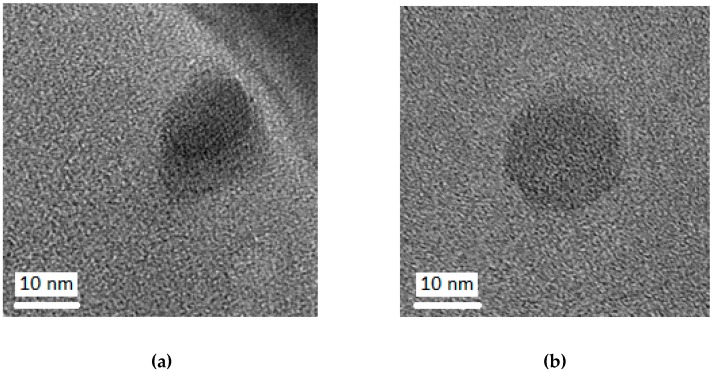
TEM images of (**a**) blank NCs and (**b**) BZP3-NCs.

The results obtained by photon correlation spectroscopy were confirmed by morphological analysis of particles by TEM. Blank NCs (Figure 2a) and BZP3-loaded NCs (Figure 2b) ranged in size between 200–500 nm, exhibiting a spherical shape.

The production of a semi-solid formulation containing BZP3-NCs was successfully achieved. The developed semi-solid sunscreen was of white and homogeneous appearance, with a good spreadability. Its effectiveness as sunscreen was calculated as a measure of the SPF, which was determined in comparison to carrot oil and to the developed aqueous dispersions of NCs (blank NCs and BZP3-NCs) (Table 3).

Carrot oil has been used as the inner core of nanocapsules to solubilize benzophenone-3 and has been demonstrated to have some sun protection effect on its own. The oil has been recently formulated as oil-based cosmetic emulsions, for which the authors have recorded a SPF value of 6.92 [29]. Nanocapsules alone also demonstrated capacity to absorb UV light, while a synergistic effect could be shown when combining all three ingredients i.e., the oil, NCs, and the active ingredient (BZP-3). SPF of BZP3 is nevertheless dependent on its concentration in the formulation [12]. The incorporation of BZP3-NCs into the semi-solid formulation slightly increased the SPF values from 8.64 to 8.84.

**Figure 3:**
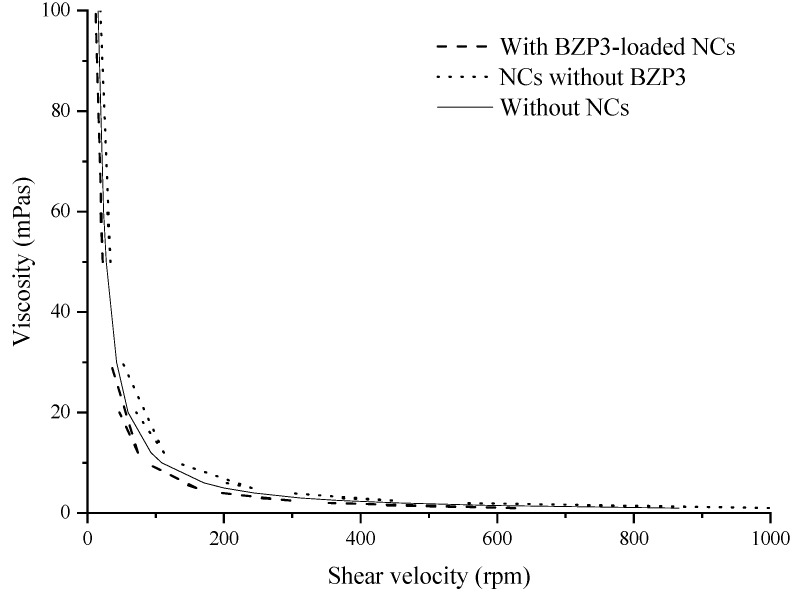
Variation of the viscosity as a function of shear rate of blank semi-solid formulation (without NCs), semi-solid formulation with blank NCs (NCs without BZP3) and semi-solid sunscreen (with BZP3-loaded NCs).

The developed semi-solid sunscreen showed a decrease in viscosity with increasing shear velocity (Figure 3), a behavior that is typical of non-Newtonian pseudoplastic formulations commonly used in topical applications. These formulations are characterized by their return to the initial viscosity, when tension (shear velocity) is reduced or interrupted. The topical administration of NCs incorporated in a semi-solid formulation combines the advantage of prolonging the residence time of BZP3 onto the skin and offering an opportunity for the sustained release of the active. The developed semi-solid sunscreen has additional attributes such as improved spreadability and greater uniformity of nanoparticle distribution when applied onto to the skin.

The semi-solid sunscreen was submitted to the centrifugation test for the assessment of its stability, wherein it showed no risk of phase separation over time. No precipitation was visible over a period of 7 days. The recorded pH values at time zero and after 7 days slightly decreased from 7.0 to 6.8, respectively, which again confirm the feasibility for topical application as skin is characterized by a acid mantle of pH 4–6 [30].

The HET-CAM test is widely used to evaluate the potential eye irritation of raw materials [20,21,22]. This method consists of the evaluation of irritation potential by the application of the product onto the chorion-allantoic membrane (CAM) of chicken eggs, and determining hyperemia, hemorrhage, and coagulation/opacity on the 10th day of incubation.

**Figure 4:**
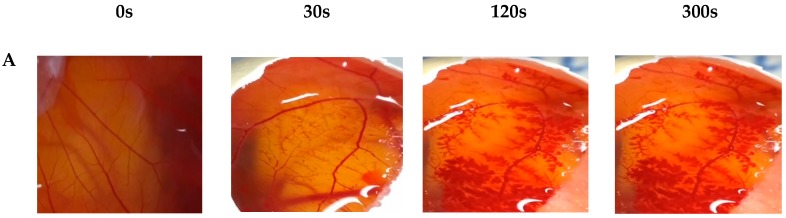

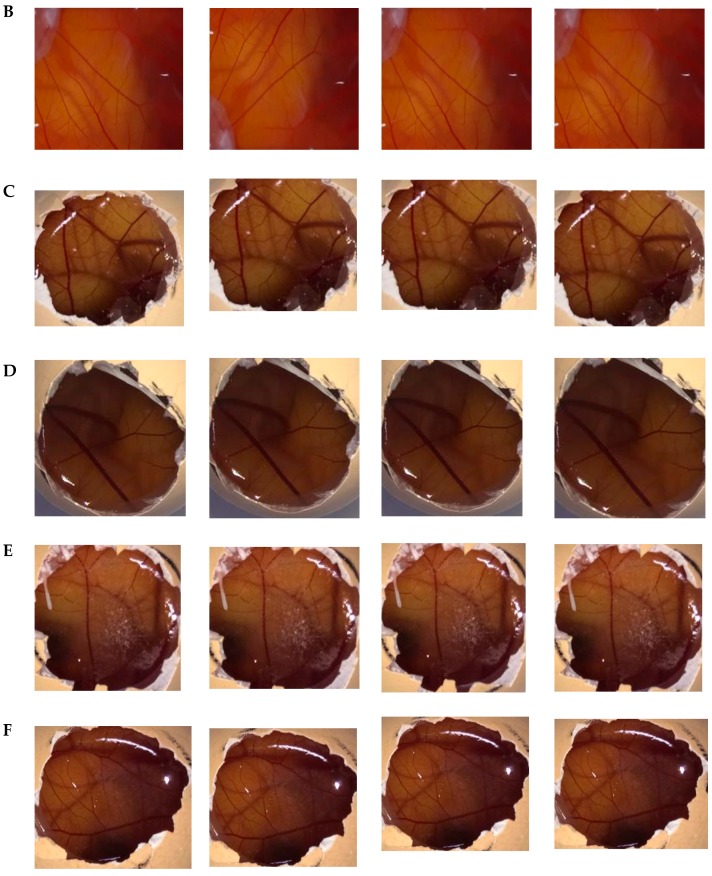
Representative stereomicrographs of Chorioallantoic Membrane (CAM) after application of: (**A**) 0.1 M sodium hydroxide (NaOH) solution, (**B**) 0.9 wt% saline solution, (**C**) polymeric nanocapsules (NCs), (**D**) polymeric nanocapsules loading benzophenone-3 (BZP3-NCs), (**E**) blank semi-solid formulation (without NCs), and (**F**) semi-solid sunscreen formulation (with BZP3-NCs).

Eyelids are a sensitive region commonly in contact with cosmetic sunscreens, and it is many times neglected in the irritation studies of sunscreens. Together with the fact that BZP3 is aggressive to skin and eye, we have carried out the ocular irritation study of our BZP3-loaded NCs in a HET-CAM assay. HET-CAM is an easy-to-use and highly sensitive in vitro method for predicting the ocular irritation effect of cosmetic products. HET-CAM is a reliable screening assay for the non-irritating potential of colloidal systems such as nanoparticles. The positive control sample of 0.1 M of NaOH is considered a strong irritant, while the negative 0.9 wt% of NaCl shows no irritation [21,31]. Based on the irritation scores of the control formulations, the analyzed formulations were classified as non-irritating ingredients, since no vascular events were observed during the 300 s (Figure 4).

**Figure 5:**
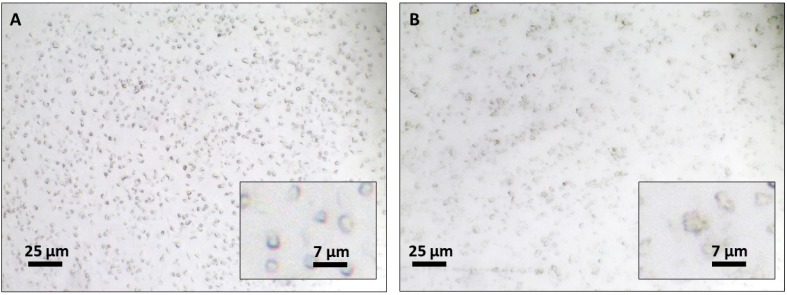
Optical microscopy images of the semi-solid formulation containing blank NCs (without benzophenone-3) (**A**) and containing the benzophenone-3-loaded NCs (BZP3-NCs) (**B**).

Both the semi-solid formulations, containing blank NCs (without benzophenone-3) in Figure 5A and containing the benzophenone-3-loaded NCs (BZP3-NCs) in Figure 5B were analyzed by optical microscopy to confirm that NCs remained individualized within the semi-solid network (Figure 5). Uniformily distributed spherical shaped nanocapsules of approximately 3 µm were clearly visible in both the cases.

## 4. Conclusions

The results reported in this work show that benzophenone-3 was easily formulated in carrot oil containing poly-ε-caprolactone nanocapsules, with predictable stability for application in sunscreens and with no cytotoxicity against fibroblasts cells. The developed semi-solid sunscreen formulation containing the benzophenone-3-loaded NCs exhibited appropriate SPF values, without irritation potential in HET-CAM test. Nanocapsules remained individualized within the semi-solid network without tendency for aggregation over time.

## Figures and Tables

**Table 1:** Composition of polymeric nanocapsules (NCs).

Formulation	F1 * [wt.%]	F2 ** [wt.%]
Organic Phase		
Poly(ε-caprolactone)	0.017	0.017
Carrot oil	0.005	0.005
Benzophenone-3	0.0005	-
Acetone	10	10
Aqueous phase		
Pluronic^®^ F68	0.02	0.02
Distilled water (q.s.)	20 mL	20 mL

* F1—Formulation no. 1 containing BZP3; ** F2—Formulation no. 2 without BZP3.

**Table 2:** The values of Z-Ave, PDI and ZP for polymeric nanocapsules.

Formulation	Z-Ave [nm] ± SD	PDI [−]± SD	ZP [mV] ± SD
NCs-BZP3	418.60 ± 56.28	0.366 ± 0.062	+11.30 ± 0.79
NCs	287.43 ± 13.60	0.189 ± 0.079	+6.11 ± 0.87

**Table 3:** Sun protection factor (SPF) of carrot oil, blank NCs, BZP3-NCs, and semi-solid sunscreen.

Carrot Oil	Blank NCs	BZP3-NCs	Semi-Solid Sunscreen
6.80	6.84	8.64	8.84

## References

[1] Tampucci S., Burgalassi S., Chetoni P., Monti D. (2018). Cutaneous permeation and penetration of sunscreens: Formulation strategies and in vitro methods. Cosmetics.

[2] Serpone N., Dondi D., Albini A. (2007). Inorganic and organic UV filters: Their role and efficacy in sunscreens and suncare products. Inorg. Chim. Acta.

[3] Gabros S., Zito P.M. (2019). Sunscreens and Photoprotection. StatPearls [Internet].

[4] Suh H.W., Lewis J., Fong L., Ramseier J.Y., Carlson K., Peng Z.H., Yin E.S., Saltzman W.M., Girardi M. (2019). Biodegradable bioadhesive nanoparticle incorporation of broad-spectrum organic sunscreen agents. Bioeng. Transl. Med..

[5] Severino P., Moraes L.F., Zanchetta B., Souto E.B., Santana M.H. (2012). Elastic liposomes containing benzophenone-3 for sun protection factor enhancement. Pharm. Dev. Technol..

[6] Cefali L.C., Ataide J.A., Eberlin S., da Silva Goncalves F.C., Fernandes A.R., Marto J., Ribeiro H.M., Foglio M.A., Mazzola P.G., Souto E.B. (2018). In vitro SPF and Photostability Assays of Emulsion Containing Nanoparticles with Vegetable Extracts Rich in Flavonoids. AAPS PharmSciTech.

[7] Souto E.B., Anselmi C., Centini M., Muller R.H. (2005). Preparation and characterization of n-dodecyl-ferulate-loaded solid lipid nanoparticles (SLN). Int. J. Pharm..

[8] Xia Q., Saupe A., Muller R.H., Souto E.B. (2007). Nanostructured lipid carriers as novel carrier for sunscreen formulations. Int. J. Cosmet. Sci..

[9] Wawrzynczak A., Feliczak-Guzik A., Nowak I. (2016). Nanosunscreens: From nanoencapsulated to nanosized cosmetic active forms. Nanobiomaterials in Galenic Formulations and Cosmetics.

[10] Krause M., Frederiksen H., Sundberg K., Jørgensen F., Jensen L., Nørgaard P., Jørgensen C., Ertberg P., Juul A., Drzewiecki K. (2018). Presence of benzophenones commonly used as UV filters and absorbers in paired maternal and fetal samples. Environ. Int..

[11] Niakousari M., Damueh M., Gahruie H.H., Bekhit A., Greiner R., Roohiejad S. (2018). Conventional emulsions. Emulsion-Based Systems for Delivery of Food Active Compounds: Formation, Application, Health and Safety.

[12] Gilbert E., Roussel L., Serre C., Sandouk R., Salmon D., Kirilov P., Haftek M., Falson F., Pirot F. (2016). Percutaneous absorption of benzophenone-3 loaded lipid nanoparticles and polymeric nanocapsules: A comparative study. Int. J. Pharm..

[13] Marcato P.D., Caverzan J., Rossi-Bergmann B., Pinto E.F., Machado D., Silva R.A., Justo G.Z., Ferreira C.V., Duran N. (2011). Nanostructured polymer and lipid carriers for sunscreen. Biological effects and skin permeation. J. Nanosci. Nanotechnol..

[14] Paese K., Jager A., Poletto F.S., Pinto E.F., Rossi-Bergmann B., Pohlmann A.R., Guterres S.S. (2009). Semisolid formulation containing a nanoencapsulated sunscreen: Effectiveness, in vitro photostability and immune response. J. Biomed. Nanotechnol..

[15] Siqueira N.M., Contri R.V., Paese K., Beck R.C., Pohlmann A.R., Guterres S.S. (2011). Innovative sunscreen formulation based on benzophenone-3-loaded chitosan-coated polymeric nanocapsules. Skin Pharmacol. Physiol..

[16] Teixeira Z., Dreiss C.A., Lawrence M.J., Heenan R.K., Machado D., Justo G.Z., Guterres S.S., Duran N. (2012). Retinyl palmitate polymeric nanocapsules as carriers of bioactives. J. Colloid Interface Sci..

[17] Batista-Duharte A., Jorge Murillo G., Pérez U.M., Tur E.N., Portuondo D.F., Martínez B.T., Téllez-Martínez D., Betancourt J.E., Pérez O. (2016). The Hen’s Egg Test on Chorioallantoic Membrane: An Alternative Assay for the Assessment of the Irritating Effect of Vaccine Adjuvants. Int. J. Toxicol..

[18] Piñón-Segundo E., Llera-Rojas V.G., Leyva-Gómez G., Urbán-Morlán Z., Mendoza-Muñoz N., Quintanar-Guerrero D. (2018). The emulsification-diffusion method to obtain polymeric nanoparticles: Two decades of research. Nanoscale Fabrication, Optimization, Scale-up and Biological Aspects of Pharmaceutical Nanotechnology.

[19] Santos E.P., Freitas Z.M., Souza K.R., Garcia S., Vergnanini A. (1999). In vitro and in vivo determinations of sun protection factors of sunscreen lotions with octylmethoxycinnamate. Int. J. Cosmet. Sci..

[20] Sanchez-Lopez E., Egea M.A., Cano A., Espina M., Calpena A.C., Ettcheto M., Camins A., Souto E.B., Silva A.M., Garcia M.L. (2016). PEGylated PLGA nanospheres optimized by design of experiments for ocular administration of dexibuprofen-in vitro, ex vivo and in vivo characterization. Colloids Surf. B Biointerfaces.

[21] Fangueiro J.F., Calpena A.C., Clares B., Andreani T., Egea M.A., Veiga F.J., Garcia M.L., Silva A.M., Souto E.B. (2016). Biopharmaceutical evaluation of epigallocatechin gallate-loaded cationic lipid nanoparticles (EGCG-LNs): In vivo, in vitro and ex vivo studies. Int. J. Pharm..

[22] Araujo J., Vega E., Lopes C., Egea M.A., Garcia M.L., Souto E.B. (2009). Effect of polymer viscosity on physicochemical properties and ocular tolerance of FB-loaded PLGA nanospheres. Colloids Surf. B Biointerfaces.

[23] De Brum T.L., Fiel L.A., Contri R.V., Guterres S.S., Pohlmann A.R. (2015). Polymeric Nanocapsules and Lipid-Core Nanocapsules Have Diverse Skin Penetration. J. Nanosci. Nanotechnol..

[24] Davaeifar S., Modarresi M.H., Mohammadi M., Hashemi E., Shafiei M., Maleki H., Vali H., Zahiri H.S., Noghabi K.A. (2019). Synthesizing, characterizing, and toxicity evaluating of Phycocyanin-ZnO nanorod composites: A back to nature approaches. Colloids Surf. B Biointerfaces.

[25] Utsunomiya H., Hiraishi R., Kishimoto K., Hamada S., Abe S., Bekki Y., Kamemura N. (2019). Cytotoxicity of benzophenone-3, an organic ultraviolet filter, caused by increased intracellular Zn^2+^ levels in rat thymocytes. Chem. Biol. Interact..

[26] Doktorovova S., Kovacevic A.B., Garcia M.L., Souto E.B. (2016). Preclinical safety of solid lipid nanoparticles and nanostructured lipid carriers: Current evidence from in vitro and in vivo evaluation. Eur. J. Pharm. Biopharm..

[27] Doktorovova S., Souto E.B., Silva A.M. (2014). Nanotoxicology applied to solid lipid nanoparticles and nanostructured lipid carriers—A systematic review of in vitro data. Eur. J. Pharm. Biopharm..

[28] Rigon R.B., Goncalez M.L., Severino P., Alves D.A., Santana M.H.A., Souto E.B., Chorilli M. (2018). Solid lipid nanoparticles optimized by 2^2^ factorial design for skin administration: Cytotoxicity in NIH3T3 fibroblasts. Colloids Surf. B Biointerfaces.

[29] Singh S., Lohani A., Mishra A.K., Verma A. (2019). Formulation and evaluation of carrot seed oil-based cosmetic emulsions. J. Cosmet. Laser.

[30] Ohman H., Vahlquist A. (1994). In vivo studies concerning a pH gradient in human stratum corneum and upper epidermis. Acta Derm. Venereol..

[31] McKenzie B., Kay G., Matthews K.H., Knott R.M., Cairns D. (2015). The hen’s egg chorioallantoic membrane (HET-CAM) test to predict the ophthalmic irritation potential of a cysteamine-containing gel: Quantification using Photoshop(R) and ImageJ. Int. J. Pharm..

